# Teacher beliefs, personal theories and conceptions of assessment literacy—a tertiary EFL perspective

**DOI:** 10.1186/s40468-022-00158-5

**Published:** 2022-05-02

**Authors:** Muhammad Wasim Latif, Arzoo Wasim

**Affiliations:** Jubail University College, Royal Commission Colleges & Institutes, Al Jubail, Saudi Arabia

**Keywords:** Language assessment literacy, Assessment beliefs, Conceptions, Teacher professional development

## Abstract

The purpose of this study was to pursue an enhanced understanding of teacher assessment literacy by investigating tertiary EFL practitioners’ assessment-related personal theories, conceptions and beliefs. The study was based on sociocultural theory as a theoretical framework and informed by interpretivism philosophical underpinnings. Twelve teachers from three tertiary educational institutions in the Eastern province of Saudi Arabia participated in semi-structured interviews. The data were analysed employing a thematic analysis approach. The findings revealed diversity, complexity and uncertainty in teacher beliefs and personal theories related to various aspects of assessment and testing. The findings also provided deeper insights into the role of contextual and institutional dynamics that influence teachers’ assessment-related decision-making process. These findings have implications for teacher education and professional development programmes in terms of assessment policy, procedures and practice.

**Supplementary Information:**

The online version contains supplementary material available at 10.1186/s40468-022-00158-5.

## Introduction

Recent times have observed a revolutionary transformation in classroom instructional practices in EFL/ESL contexts worldwide against the backdrop of increased expectations for teachers to drive their learners towards ever-higher accomplishment standards. These standards revolve around the goals of preparing learners for lifelong learning skills to meet the challenges of the current century (Hopfenbeck, 2018; Nguyen & Walker, 2016; Siarova et al., 2017). Helping students succeed requires that teachers be able to develop assessment tasks that evaluate learner higher-order thinking skills and broader knowledge (Darling-Hammond & Adamson, 2013). In doing so, teachers are expected to align their assessment practices with a learning-oriented assessment culture driven by a well-structured formative assessment system (Inbar-Lourie, 2008b; Shepard, 2013). To ensure the effective utilization of language assessments for learning purposes, whether they are formative or summative, it is essential that a language teacher understands the main principles of a sound assessment.

According to Inbar-Lourie (2008a), a language teacher’s language assessment literacy is defined as his or her sound understanding of language learning theories and classroom assessment practices and his or her ability to utilize this knowledge to gauge and improve student learning by employing various assessment methods and strategies (also see Davies, 2008; Fulcher, 2012; Scarino, 2013; Scarino, 2017; Taylor, 2009; Yan & Fan, 2020). Language teachers’ beliefs and conceptions that underpin their conceptualization of the various aspects of the assessment process are one essential element of their assessment literacy (Scarino, 2013). The literature shows that comprehending teachers’ assessment beliefs and conceptions regarding their role in the assessment process is key to implementing assessment reform policies (Barnes et al., 2017). However, the way that language teachers’ assessment beliefs relate to their assessment literacy and professional development needs has rarely been examined in EFL/ESL contexts in general and in the Middle East in particular. Given that an appropriate level of assessment literacy is sine qua non for every teacher’s professional repertoire and teachers’ beliefs and conceptions are vital elements of their assessment literacy, the present study investigates how language teachers view different aspects of assessment and testing and how their belief systems work. We hope that comprehending teachers’ assessment beliefs and personal theories that inform their assessment practices can help us better understand teachers’ language assessment literacy in the tertiary EFL context of Saudi Arabia.

## Literature review

### Language assessment literacy

Language assessment literacy (henceforth LAL) generally refers to a repertoire of competences, knowledge and understanding of the use of varied assessment methods and strategies and the application of this understanding to the selection and use of appropriate assessment tools when needed. LAL makes an individual capable of understanding, assessing and constructing language test questions, analysing them and making suitable pedagogical decisions based on assessment outcomes (Coombe et al., 2020; Inbar-Lourie, 2008a). Additionally, being a social and co-constructed phenomenon, LAL requires teachers to have the ability to understand and critically evaluate the role and function of assessment practices in terms of their impact and the placement of teacher learning opportunities in a specific sociocultural, political, educational and philosophical context (Coombe et al., 2020; Fulcher, 2012; Levi & Inbar-Lourie, 2020; O’Loughlin, 2013; Scarino, 2017; Yan & Fan, 2020). Moreover, recent theoretical discussions about LAL argue that it is essential for teachers to have self-awareness by exploring and appraising their own beliefs, preconceptions and understanding regarding their own knowledge, practices and ethical standards that shape and guide their assessment-related “conceptualizations, interpretations, judgments and decisions” (Scarino, 2013, p. 309). Considering the wide-ranging competency-based scope of LAL, language teachers are required to have a high level of professionalization.

The LAL field has recently attracted scholarly discussion. Considerable research has been conducted to investigate the various aspects of LAL. For instance, LAL has been explored as a concept, in general (e.g. Coombe et al., 2012; Inbar-Lourie, 2008a; Kremmel et al., 2018; Taylor, 2013; Xu & Brown, 2016); in terms of teachers’ assessment knowledge and skills (e.g. Al-Bahlani, 2019; Jawhar & Subahi, 2020; Latif, 2021; Rauf & McCallum, 2020); with regard to teachers’ perceived assessment training needs (e.g. Tsagari & Vogt, 2017; Vogt et al., 2020; Zulaiha & Mulyono, 2020); regarding the importance of the need for LAL development among different stakeholders (e.g. Kremmel & Harding, 2020; O’Loughlin, 2013); and in relation to teacher assessment conceptions (e.g. Brown et al., 2019; Giraldo, 2019). However, the area of LAL identified by Scarino (2013) which relates to teachers’ “lifeworlds” revolving around their beliefs and personal theories in relation to their assessment literacy and professional development needs in specific contextual dynamics, which Inbar-Lourie (2017) terms teachers’ “local realities” has not received sufficient scholarly attention.

### Research on teacher assessment beliefs, conceptions and personal theories

The term “belief” is defined as “an individual’s judgment of the truth or falsity of a proposition” (Pajares, 1992, p. 316). Individuals’ personal theories relate to their interpretation of the various aspects of a particular phenomenon based on their understanding and worldview that they develop after having observed and experienced varied realities of life (Buchanan, 2015). A conception refers to beliefs and knowledge unified into a single construct providing a framework for the description of teachers’ overall understanding and perceptions of assessment (Barnes et al., 2017; Thompson, 1992). Although these three terms “beliefs”, “personal theories” and “conceptions” have different meanings, they are interconnected. In the present study, these constructs refer to a tertiary EFL practitioner’s beliefs, perceptions and understanding of the various aspects of the language assessment process and teacher assessment literacy. Our main focus is on the exploration of beliefs, and we also explore other constructs related to beliefs.

In the past two decades, although a number of scholars have investigated teachers’ beliefs about classroom assessment practices in the school context (e.g. Barnes et al., 2015; Davison, 2004; McMillan & Nash, 2000; Remesal, 2011), there is a dearth of scholarship on EFL/ESL teachers’ assessment beliefs and conceptions in relation to their assessment literacy and professional development needs at the tertiary level, especially in the context of the Middle East.

Brown (2008) identifies four major assessment-related conceptions and beliefs held by teachers. Three of these concern the purpose of assessment in terms of school accountability; students’ and teachers’ accountability; and pedagogical improvement. The fourth has no connection with assessment purposes; it refers to the use of assessment for administrative reasons only, so it has no relevance to student learning based on teachers’ work.

The literature indicates that teachers feel some uncertainty and hold conflicting beliefs and conceptions regarding assessment purposes, methods and roles (e.g. McMillan & Nash, 2000; Remesal, 2011; Xu & Liu, 2009). In their critical study in the EFL context in China, Xu and Liu (2009) conclude that teachers generally experience various conflicts in terms of the purpose and function of assessment in the whole assessment process; however, they seem to perceive traditional assessments such as summative tests as the most appropriate assessment tools to measure learner achievement rather than alternative or innovative assessment methods. These conclusions are in contrast to the findings of McMillan and Nash’s (2000) questionnaire-based study which indicated teacher beliefs supporting alternative or innovative assessments.

The literature also reveals that teacher beliefs about assessment practices are formed under the influence of specific institutional, cultural and educational policy dynamics, highlighting the integral relationship between assessment and its social context (e.g. Cheng et al., 2004; McNamara, 2001; Rogers et al., 2007; Vogt et al., 2020). In a recent mixed-method study, Vogt et al. (2020) investigate the role of sociocultural dynamics in the assessment process by exploring Greek and German English language teachers’ beliefs and insights about language assessment and their professional development needs. The findings revealed that although teachers generally conceptualize various elements of assessment in a somewhat similar manner, their assessment beliefs and perceptions of PD needs are complex, multi-dimensional and varied due to the varying sociocultural dynamics of a particular educational setting. This underscores the significance of the connection between assessment and its social context. Teachers’ diverse beliefs reflecting their varied cultural and societal backgrounds have an impact on their teaching and assessment practices (Brown et al., 2011). This argument is in line with the findings of some other studies (e.g. Berry et al., 2019; Ferretti et al., 2021; Troudi et al., 2009). In the Italian EFL context, Ferretti et al. (2021) recently studied teachers’ assessment beliefs in the context of long-distance learning approach due to the COVID-19 crisis. Based on the analysis of questionnaire data, they concluded that teachers experienced some confusion and uncertainty in terms of their understanding of the assessment in general and assessment purpose and methods in particular in the given long-distance learning environment. The findings indicated that teachers believed in summative assessments as ‘true’ assessments, but they felt that these assessments could not be effective in the crisis situation, as these assessments clash with established assessment norms and practices.

In the context of the Middle East, there has been no substantial research on language teachers’ assessment beliefs, conceptions, or assessment practices in relation to their assessment literacy (but see Firoozi et al., 2019; Hidri, 2016). In their interview-based study in the Iranian school context, Firoozi et al. (2019) conclude that there is a need for change in language teachers’ current assessment-related perceptions if new assessment policies that aim to shift from traditional testing culture to a performance-based assessment system are to be implemented successfully. Hidri (2016), on the other hand, studies assessment conceptions of secondary school and university teachers in an EFL context of Tunisia employing the teachers’ conceptions of assessment (TCoA) inventory (Brown, 2006). The results indicate a significant relationship between teachers’ conceptions regarding the use of assessment for *accountability* as well as *improvement* purposes.

The above review of the literature indicates a paucity of scholarship on teachers’ assessment literacy in terms of their assessment beliefs, conceptions and personal theories in tertiary EFL contexts in general and the Arab world in particular. The present study intends to bridge this gap in the literature. We hope that this research will be helpful in understanding the concept of language teachers’ assessment literacy in a particular socially and culturally contextualized setting.

## Theoretical framework

The study is positioned within a wide-ranging interpretive philosophical framework. Theoretically, it is informed by Vygotsky’s (1978) sociocultural theory, which recognizes the central place and role of sociocultural dynamics in the process of language and language assessment literacy development. According to McNamara (2001), there is an inseparable connection between assessment and its social context. Every context has its own distinct institutional and educational policy dynamics, “which contribute to shaping the preconceptions about assessment purposes, constructs, methods and judgements that teachers bring to the process of developing assessment literacy” (Scarino, 2013, p. 312). Given the assessment challenges confronting EFL/ESL practitioners in the context of conceptual shifts in contemporary language learning theories and assessment practices resulting in the continuous evolution of the notion of language teacher assessment literacy, it is important to explore EFL practitioners’ beliefs, preconceptions and understanding that shape their conceptualizations, interpretations, decisions and judgement in assessment.

### Research questions

1. How do tertiary EFL practitioners generally view the assessment and testing process?
2. What are tertiary EFL practitioners’ beliefs about classroom assessment?
3. What are tertiary EFL practitioners’ views and beliefs about assessment methods, strategies and procedures?
4. How do tertiary EFL practitioners view the procedures related to assessment quality standards?

## Methodology

An exploratory methodology was deemed appropriate to investigate the research questions because of its alignment with the study’s theoretical background.

### Participants and the context

A total of twelve tertiary EFL practitioners were selected to participate in semi-structured interviews. They were working in three higher education institutes (2 public; 1 private) located in the Eastern province of Saudi Arabia.

Of the total 12 participants, the majority were males (9). All participants were 35–55 years old (Table 1). The selection of the participants was based on a purposive sampling technique. According to Cresswell and Plano Clark (2018), a researcher employing this technique intends to identify and select persons or groups of persons who are capable in and up-to-date with a phenomenon of interest, and the purpose is to elicit the most information possible regarding the question being investigated.

**Table 1:** Participants’ background information

		n = 12
Nationality	American (Karen, James)	2
	Irish (Nathon)	1
	South African (Daniel, Luan)	2
	Nigerian (Ahmad, Emem)	2
	Filipino (Alfonso and Louise)	2
	Saudi (Turki)	1
	Jordanian (Ahyam)	1
	Pakistani (Tahira)	1
Educational background	Doctoral degree	4
	Master’s degree	6
	Bachelor degree (English major)	2
Teaching experience	0–5 years	1
	6–10 years	4
	11–15 years	4
	16–20 or more than 20 years	3
Professional qualification/training in assessment and testing	Basic	7
	Advanced	3
	None	2

### Instrument and procedures

In-depth interviewing is an essential part of qualitative research. In the present study, the purpose of using semi-structured interviews was to explore teachers’ beliefs, conceptions and personal theories of language assessment that inform their assessment practices in conjunction with their knowledge base. To generate ideas for the interview questions, four pilot interviews were conducted. During the administration of the interview sessions, the content and sequence of the questions was flexible; interviews were “tailored to each individual interviewee and the responses given, with prompts and probes” (Cohen et al., 2018, p. 942).

Since the purpose of the study was to comprehend the patterned meanings of the phenomenon under investigation through data interpretations based on the in-depth comprehension of the participants’ statements, data were analysed using a thematic analysis approach that involved data reduction in the form of codes, categories and themes. The coding process started with the colour coding of the transcribed interview data, which involved careful word-by-word reading of the text. Statements reflecting similarities, differences, regularities, irregularities and oddness were selected and coded. This approach was in line with Saldana (2015) recommendation that carefully noting repetitive patterns and consistencies in human actions and words is important for identifying emerging categories and themes. Then, drawing on Cohen et al. (2018) and Creswell and Creswell (2018), the whole dataset was separated into manageable parts in the form of codes, categories and sub-categories (open coding), and then, these codes, categories and sub-categories were mixed to explore central categories (axial coding). In the last stage (selective coding), the core categories were identified and their connection with other categories was examined, which led us to generate themes for our narrative. Being aware of the cyclical and iterative nature of this coding process, we ensured constant back-and-forth movement between the entire data set in search of the emerging themes identifying with Braun and Clarke (2006), who suggested that researchers need to continuously review, revise and refine codes, emerging categories and themes.

## Findings

The analysis of interview data revealed a number of significant aspects of tertiary EFL teachers’ beliefs and perceptions regarding the various aspects of their assessment literacy. Table 2 shows the themes and sub-themes, which are discussed in detail in the following sections, supported by extracts from interviews.

**Table 2:** Tertiary EFL practitioners’ assessment beliefs

Themes	Sub-themes
1. Beliefs about assessment and testing in general	a) Characteristics of good assessment
	b) Responsibility for design of assessment
2. Beliefs about classroom assessment	a) Activities considered classroom assessment
	b) Use of classroom assessment activities
	c) Reasons for using classroom assessment
	d) Effective activities in different classroom contexts
3. Beliefs about assessment methods, strategies and procedures	a) Methods for assessing macro skills
	b) Approach to grading summative and formative assessment
	c) Interpretation: criterion-referenced preferred
	d) Communicating results to different stakeholders
4. Beliefs about assessment quality standards	a) Formative assessment system-based procedures
	b) Pre and post-assessment analysis and evaluation
	c) Personal quality checks related to self-designed tests

### Beliefs about assessment and testing in general

The findings of this section centre on two sub-themes, as shown in Table 2. When asked about the characteristics of a good assessment, all participants reported validity, reliability and fairness as the main characteristics. Four participants believed that a quality assessment, in addition to being valid, reliable and fair, should also be authentic and context-based. Two participants believed that a good assessment is varied and motivating and has consequential relevance. These findings reflect the multiplicity of participants’ perceptions and interpretations of a good assessment.

Likewise, when the participants were asked about their views regarding the design and execution of assessment processes, mixed responses were provided. For instance, half of the participants believed that at the institutional level, assessments should be designed internally by the teachers of the course, as is evident in one participant’s response:

… I believe that a good assessment should be based on the knowledge of the teacher because he/she understands the dynamics of the institutional as well as classroom context … he/she is the one who gave the instruction based on different activities knowing the students’ level … (Alfonso)

Four participants believed that a good assessment is one that is designed in-house. However, regarding responsibility for assessment writing, one of these participants believed that it is an institutional test committee comprising only specialists in test writing that should be responsible for test development. The other three participants reported that this responsibility depends on the purpose of the assessment. If the purpose is formative, the teacher of the course should take the responsibility, but if the purpose is summative, the test committee should take responsibility. Two participants believed in the parallel use of internationally recognized standardized tests such as TOEFL/IELTS and in-house developed tests. Although there was some disagreement on whether it is an institutional test committee or teachers who are responsible for the design of assessments, a majority of the participants seemed to believe in the major role and responsibility that the teachers should have in the design and execution of assessment processes. This finding indicates the importance of teacher preparation in assessment-related skills and knowledge.

### Beliefs about classroom assessment

The findings in this section revolve around four sub-themes, which are presented below.

#### Activities considered classroom assessment

When asked about the definition of classroom assessment, the participants expressed diverse views. However, almost all believed that the main purpose of classroom assessment is to monitor the progress of the learning process, which can be done in many ways. For instance, four participants believed that classroom assessment entails pre-lesson diagnostic assessment done in oral or written form to check students’ knowledge, activities done during the lesson and progress assessments at the end of a lesson. According to three participants, classroom assessment includes all activities done in classroom that have formative and summative purposes.

On the other hand, three participants believed that the purpose of classroom assessment is to assess students’ learning inside the classroom in order to inform better instructional practices, and the classroom assessment can be in the form of

…informal checks for understanding like questions in the class all the way to more performance-type activities, such as doing a short discussion/presentation; that’s for speaking skills. And if it is writing skills, you can have short mini-assignments in the class… (Nathon)

Two participants saw classroom assessment as different from formal tests or assessments. According to them, the focus of a classroom assessment is not to assign grades; rather, it is to collect feedback that helps teachers plan their teaching practice in alignment with the needs of their learners, and this can be done using various classroom activities depending on the lesson and course objectives. The analysis of participants’ responses shows that although their views reflect a varied understanding of the concept of classroom assessment, the majority generally believed that any form of activity carried out in the classroom aiming to monitor students’ learning progress can be categorized as classroom assessment.

#### Use of classroom assessment activities

The participants’ responses relating to their beliefs and perceptions of the various classroom assessment types that they generally used to gauge students’ learning revealed that they believed in using various classroom assessments, such as self-assessment, peer-assessment, teacher-student conferences, portfolio assessment, oral presentations, reflective journals and authentic assessment, depending on the class context. When asked specifically how often they used each of these classroom assessments, participants’ responses reflected diversity in their beliefs about the importance of these assessments.

The most frequently used classroom assessments as reported by the participants were self-assessment, peer-assessment, teacher-student conferences and oral presentations. All participants reported using oral presentations because they were a course requirement. The classroom assessments used the least were portfolio assessment, reflective journals and authentic assessments. Two participants said that they did not use self-assessment at all because of the difficulties involved in using these assessments in their contexts. Half of the participants reported having no knowledge of reflective journals or authentic assessments. Their lack of clarity is reflected in the response of one participant to a question regarding authentic assessment:

…Well, an authentic assessment is a usual assessment like exams, tests, quizzes in a formal situation…maybe involving all students…this is how I understand what an authentic assessment is… (Ahmad)

Lack of clarity about its meaning was expressed by another participant:

I don’t know what an authentic assessment really means… but is it an assessment which is something reliable… this is what I feel...yes, I do; if it doesn’t have meaning more than this (Louise)

Likewise, one-third of the participants were not clear about portfolio assessment or teacher-student conferences. Four participants reported that although they believed that portfolio assessments and reflective journals have great pedagogical importance, they did not use them as there was no institutional policy about them, and they also had time constraints because of a heavy teaching workload.

#### Reasons for the use of classroom assessment activities

The participants identified diverse reasons that influenced their decision to use or not use a particular classroom assessment activity. For half of the participants (6), the skill/course content or course learning outcomes motivated them to use classroom assessments. On the other hand, three participants reported that their decision to use any classroom assessment was based on students’ level. One participant explained why some of the assessment types were not appropriate:

I’m not going to ask the weak students to have reflective journals because it would be a waste of time …they are not going to do it…similarly, you can’t use self-assessments if you mostly have weak students in the class…unless you have a mixed-ability class where you have more options to explore (Ahyam)

For two participants, the macro-level social context and micro-level institutional context also influenced their decision regarding the use of these classroom assessments. One participant explained:

Peer assessment - I don’t use it much, as there is a lot of face saving here…the students don’t assess others’ work neutrally… I think it has something to do with the culture itself (Ahmad)

One participant reported that he used classroom assessments taking into consideration the time of the semester, that is, the beginning, middle or end. He believed that higher-order assessments such as portfolio assessment, peer-assessment and teacher-student conferences should be used towards the end of the semester only once the students are ready and have been exposed to and trained at selected response and simple constructed response item-based assessments.

#### Effective activities in different classroom contexts

The analysis of participants’ responses reveals that all participants believed that the classroom assessments such as self-assessment, peer-assessment, teacher-student conferences, oral presentations, reflective journals, portfolios and authentic assessments are useful and can be used with effective results. However, participants had diverse views about the classroom assessment activities that they found most useful. For them, the most useful classroom assessments are as follows: authentic assessment (6), peer-assessment (3), teacher-student conferences (2) and oral presentations (1). For assessing students’ receptive skills, the majority (7) believed that self-assessment and peer-assessment can be used with the most effective results; three participants advocated the use of teacher-student conferences and two supported informal questioning in class. Regarding the assessment of productive skills, almost all participants believed that all of the classroom assessments mentioned above can generally be used with good results; however, the majority believed that self-assessment, peer-assessment and authentic assessments are the most useful for the assessment of writing skills and that oral presentations and authentic assessments are the most effective for the assessment of speaking skills. One participant believed that all of the abovementioned assessments are useful for assessing both receptive and productive skills, but there is a hierarchy. He expressed his views in these words:

Well, I believe that all these classroom assessment types are extremely useful and can be used, but they should go in progression…it depends on what time/part of the semester it is; what level of students you are dealing with… (Daniel)

### Beliefs about assessment methods, strategies and procedures

The findings of this section revolve around four sub-themes as follows.

#### Methods for assessing macro skills

The first sub-theme related to participants’ preferred methods for assessing receptive and productive skills reveals participants’ generally mixed beliefs regarding different methods. In the literature, assessment methods are classified into three broad categories: 1. selected-response methods (SRM) (e.g. matching (fill-in with lists) or multiple choice (sentence-completion, gap-fill etc.) and discrimination (same/different, true-false etc.); 2. constructed-response methods (CRM) (limited production tasks and/or extended production tasks); and 3. personal-response methods (PRM) (e.g. self- and peer-assessments, conferences, portfolios) (Grabowski & Dakin, 2014; Popham, 2014).

The data analysis revealed that it was mainly a combination of SRM and CRM that participants believed in using to assess receptive skills, whereas for the assessment of productive skills, they believed in using a combination of CRM and PRM. Additionally, four participants believed that CRM and PRM can be used to assess both receptive and productive Skills. In addition to expressing their preferences for the assessment method, some participants also advocated the use of authentic or performance-based assessment tasks for both receptive and productive skills. For another participant, the selection and use of various assessment methods vary at different stages of the semester, which is reflected in these words:

Well, again...my training is taxonomy… so, I will use simple kind of assessment design, i.e. MCQs, Fill-in types of questions at the start, but as we go through, I will be putting in more of the short essay-type questions, and towards the end, I will be doing portfolios, conferencing etc... so, it’s from simple kinds of exams to higher level to creative types of assessments (Daniel)

The analysis also revealed that PRM and SRM were the least preferred methods for assessing receptive skills and productive skills, respectively.

#### Approach to grading summative and formative assessment

The second sub-theme relates to the marking of exams on productive skills (writing and speaking). The participants described different approaches depending on the assessment type, that is, formative or summative. For formative assessments, the majority (9) preferred analytic marking to holistic marking (3). On the other hand, for summative assessments, the majority (8) preferred holistic marking to analytic marking (4). One participant explained the reasons for his preference for the analytic approach to the holistic approach in the following words:

… I’m a teacher…I always go for analytic marking since I don’t mark because I want to put a grade to my students’ output…I would like to mark so I can hopefully give feedback to my students on their strengths and weaknesses. (Alfonso)

The majority of participants’ preference for the holistic marking approach for formal and summative assessment was based on certain contextual and personal reasons, such as the complexity of the institutional policy regarding feedback on formal assessments, time constraints and lack of motivation. The following extract highlights one of the participants’ concerns regarding institutional policies related to post-assessment feedback:

…here, we have certain contextual or what you call institutional constraints… for example, at the end of last semester mid-term exams, I planned to give feedback by showing the papers to the students, but the next day, there came an email from management asking teachers not to show the papers to the students. (Alfonso)

Alfonso believed that there is no point in marking exams analytically if students cannot see their exams. Another participant, Ahmad, explained the influence of time constraints: “Well, I prefer to use holistic marking because I believe holistic marking is better than analytic marking, which is more time-consuming… and that’s the big issue for us here especially with our tough schedules...” (Ahmad). When asked which marking approach they believed is the best if they had the choice, most participants advocated the use of analytic marking, as it is more objective, has clear criteria to follow and is important for feedback to address learner needs.

#### Results’ interpretation: criterion-referenced preferred

For the third sub-theme, the interpretation of assessment results, the majority of the participants showed a preference for the criterion-referenced approach. Nine out of the twelve interviewees believed in interpreting student performance in an assessment following certain predetermined or preidentified benchmarks, i.e. criterion-referenced interpretation, and two participants believed in interpreting a student’s performance in comparison with other students in the class, i.e. norm-referenced interpretation. It is important to mention that we had to explain the terms norm-referenced and criterion-referenced to eight of the twelve participants, as they were not clear about these terms. One participant who advocated criterion-referenced interpretation explained his choice:

I would like to base my explanation of the students’ results on some criteria, benchmarks, or course objectives not on the performance of others in the group. I believe that this is the way to go. *(*Alfonso)

Two interviewees reported that although they found both norm and criterion-referenced results’ interpretation useful, they believed in using criterion-referenced more than norm-referenced interpretation.

One of these participants stated the following:

…Well, if you really have to measure students’ performance in the assessment validly, you have to assess them on different days in order to see the students’ real level… my preference would be to focus on the intended learning outcome or benchmarks. *(*Ahyam)

#### Communicating results to different stakeholders

Regarding the fourth sub-theme, communicating assessment results to different stakeholders, mixed views were reported. For instance, five participants believed in students having access to computer-generated results for both summative and formative assessments, meaning that the teachers’ role in communicating results to the students should be minimal. For instance, Ahmad stated “…they check their results for the summative assessments online…this is the system here. I don’t discuss the results with them… I simply hate this…” Explaining why he hated communicating results to students, Ahmad, added that discussing results, especially on writing exams in class was a very complicated matter in his context for three reasons. First, there was a lot of face saving there…“a self-esteem issue”, so low achievers did not like their results to be reported in front of their peers. Second, reporting results takes away from teaching time and leads to class management issues. Moreover, there is no use in reporting results because students start comparing their marked writing with others’ instead of learning by focusing on their own individual performance.

In contrast, three interviewees expressed their belief in discussing results standing in front of the class and then asking every student to come to the front to get the teacher’s feedback on his or her performance. One participant believed in communicating results to the students individually and adopting a suitable approach accordingly. Three participants believed in providing detailed text-based feedback. For instance, one participant said:

I believe that students should be provided some commentary of their achievement because CEFR is not really about achievement in terms of percentages or As, Bs or Fs; rather, it is about descriptions of what you can do… (Nathon)

### Beliefs about assessment quality standards

The final theme relates to teachers’ views and perceptions of assessment quality standards. The findings in this section are categorized into three sub-themes: formative assessment system-based procedures; pre- and post-assessment analysis and evaluation; and personal quality checks related to self-designed tests.

In response to the question about what actions they thought were essential for improving assessment quality, the majority of the interviewees believed that establishing a strong formative assessment system was key to improving assessment quality. One interviewee, Ahmad, emphasized that a range of assessments should be used in the classroom aiming at learning and that teachers should not “teach materials for the sake of exams” only, which reflects his understanding of negative test washback. Another element essential for improving assessment quality that participants stressed was the need to ensure appropriate pre- and post-assessment validity and reliability checks at different stages of the assessment process. One-third of the participants believed that using authentic materials and having a system for measuring assessment quality against other internationally recognized exams were important in the process of improving overall assessment quality in an educational system.

Replying to the question about the quality procedures and actions they followed while developing their tests, the majority of participants reported relying on their self-determined assessment quality checks based on their understanding of assessment-related matters, such as assessment purpose and course learning outcomes. These personal quality checks highlight their understanding of the importance of critical thinking, context-based materials, validity and reliability factors in the assessment process.

## Discussion

The interpretation of findings revealed three major themes, which are discussed in this section. The themes are as follows: multiplicity, diversity and complexity in assessment beliefs; gaps in conceptual understanding and awareness of contemporary methods, trends and approaches to educational assessment; and belief in the improvement factor of assessment-related conceptions.

Teacher personal beliefs, conceptions and theories serve as a schema that a teacher uses to understand, interpret and deduce meanings in order to shape his or her pedagogical and assessment-related thought processes and decision-making (Borg, 2013; McMillan, 2003). According to Brown (2008), teachers’ assessment beliefs and conceptions have both cognitive and personal dimensions involving emotional predispositions. These dimensions are structured by wide-ranging interpretations regarding epistemology in general and classroom pedagogy in particular. The cognitive aspects of teacher assessment beliefs relate to what they think of the assessment process in terms of its alignment with good or bad practice. The personal dimension of teacher beliefs and conceptions, on the other hand, pertains to assessment-related emotions that teachers develop over a period of time after accruing multifaceted assessment experiences first as learners and later as teachers. These emotions may be positive or negative, deeply rooted or less deeply rooted (Phipps & Borg, 2009; Sheehan & Munro, 2017).

Apart from these cognitive and personal aspects of teachers’ belief systems regarding assessment and testing, there are macro- and micro-level contextual variables that also affect teachers’ decision-making process in their assessment practices grounded in assessment conceptions (Brown et al., 2019).

In this study, the participants’ belief that the main features of a good assessment include validity, reliability, fairness, authenticity, application of learned skills and the assessment of language production instead of language memorization reveals variations and multiplicity in terms of their interpretations. Their lack of clarity and understanding of some basic concepts, such as assessment validity, reliability and authenticity, indicate the complexity in their beliefs, on the other hand. This confusion regarding basic assessment concepts conflicts with their reported belief that an assessment should be designed in-house and that teachers should have a strong role and responsibility in the process of developing and conducting assessments. Similarly, the diversity and complexity in their views and understanding are reflected in their interpretation of classroom assessment. Although all the participants believed that the primary purpose of a classroom assessment is to monitor students’ learning progress, the impetus inspiring the use of classroom assessment activities varied. This is also reflected in their response to the question about the pedagogical significance of various classroom assessments such as self-assessment, peer-assessment, teacher-student conferences, portfolio assessment, reflective journals, oral presentations and authentic assessment. The participants’ belief in using diverse classroom assessments by virtue of their usefulness in enhancing learning in the classroom clashes with participants’ limited use and insufficient conceptual understanding of these classroom assessments. This diversity and complexity in their views, which might be attributed to complex institutional assessment policy dynamics, has implications in terms of teacher decision-making pertaining to classroom pedagogies and assessment. The findings of some previous research highlight how teachers’ assessment beliefs directly influence their decision-making process regarding assessment practices in the classroom (e.g. Al-Bakri, 2016; Hedia, 2020). The findings of Al-Bakri’s (2016) qualitative study in the tertiary EFL Omani context revealed that teachers’ beliefs largely affected their written corrective feedback strategies which were mainly inclined towards direct written corrective feedback. Hedia’s (2020) study, on the other hand, examined the connections between Tunisian EFL instructors’ beliefs and grading practices for writing courses. She also concluded that teachers’ beliefs about grading writing essays had a strong impact on their grading practices. Although both of these studies emphasize how teachers’ assessment beliefs influence their assessment practices, they also highlight certain discrepancies between professed and validated beliefs through practice. This demonstrates the multiplicity, complexity and diversity of teachers’ beliefs about the various dynamics of classroom assessment, which is echoed by the findings of some other studies (e.g. Giraldo, 2019; Latif, 2017; Rogers et al., 2007).

The literature indicates that although teachers’ assessment-related beliefs and conceptions play a strong role in inspiring their assessment practices, they cannot carry out these assessment practices in a way they would like since “they are employed within an immediate workplace community and larger social, political, and cultural contexts” (Xu & Brown, 2016, p.157). This underlines the complex character of assessment and testing influenced by distinct institutional, cultural and educational policy dynamics of the social context, which affect teachers’ assessment beliefs, knowledge base and practices (Looney et al., 2017; McNamara, 2001; Scarino, 2013).

Under the influence of micro- and macro-level contextual variables, teachers find themselves working in a “culture of certainty and compliance” marked by certain pre-identified criteria and boundaries in the form of norms, conventions, policies and rules that guide them in how and what they can and cannot practice in terms of assessments (Scarino, 2013, p. 312). In this study, the participants’ constrained approach towards feedback, grading and peer-assessment, which went against their stated beliefs, reflects their compliance with institutional policies and contextual restraints. This underlines how teachers’ assessment beliefs and personal theories are informed and how their overall role in the assessment process is shaped by multifaceted contextual factors such as their understanding of institutional policies, cultural dynamics, institutional management and/or leadership styles and classroom pedagogies. This argument is stressed by another study in the context of the Middle East (e.g.Troudi et al., 2009).

In addition to the roles of various contextual factors that contribute to the diversity and complexity in teachers’ assessment beliefs and conceptions, their varied cultural, societal, academic and professional backgrounds affect their assessment-related personal theories and beliefs. This is consistent with the findings of Brown et al.’s (2019) study, which shows that teachers’ diverse assessment beliefs and views mirror the diversity in their societal and cultural backgrounds, and their classroom pedagogical and assessment practices are influenced by this diversity (also see Rogers et al., 2007).

The second theme identified in the findings is related to gaps in tertiary EFL teachers’ conceptual understanding of some contemporary assessment methods and approaches to educational assessment. The results suggesting some of the participants had an insufficient understanding of some of the main assessment concepts, such as assessment validity, reliability, authenticity, reflective journals, portfolio assessment, and the difference between norm-referenced and criterion-referenced results, indicate gaps in teachers’ assessment knowledge base. This is consistent with the results of Al-Bahlani’s (2019) mixed-method study in the context of Oman. The findings revealed inadequacies in teachers’ knowledge of assessment principles such as validity, reliability, clarity, authenticity and practicality that they were required to know in order to evaluate assessment tasks. Somewhat in contrast to these findings, Jannati (2015) and Shim (Shim, K. N.: An investigation into teachers’ perceptions of classroom-based assessment of English as a foreign language in Korean primary education, Unpublished doctoral thesis), in two different contexts, find that while participants’ conceptual understanding of the basic principles of assessment and testing was sufficient, it was not reflected in their classroom assessment practices. This points to the complexity in teachers’ assessment literacy. In the context of this study, teachers’ insufficient assessment knowledge base has also been highlighted by recent research (e.g. Latif, 2017; Latif, 2021; Rauf & McCallum, 2020). The main factors attributable to shortfalls in language teachers’ assessment literacy were identified as a lack of strong assessment policies, institutional power and top-down management issues and lack of innovation in the delivery of teacher professional development programmes as per the demands of contemporary assessment trends. Davidson and Coombe (2019) also identify these factors as major reasons for teachers’ lack of assessment literacy in any EFL/ESL context in general and in the MENA region in particular. They argue that most LAL development initiatives for teachers primarily focus on providing assessment training generically and superficially, not holistically considering the various contextual needs and requirements of the modern era. Ideally, these initiatives must ensure that the training process entails a proper balance of “technical know-how, practical skills, theoretical knowledge, and understanding of principles, but all firmly contextualized within a sound understanding of the role and function of assessment within education and society” (Taylor, 2009, p. 27).

The third theme identified in the findings is tertiary EFL teachers’ belief in the *improvement* factor of teacher conceptions of assessment. Teachers’ conceptions of the functions of assessment, which impact the way they carry out their assessment practices, have three main aspects, i.e. *accountability*, *improvement* and *irrelevance* (Barnes et al., 2015; Brown, 2008). The accountability aspect of their conceptions derives from the notion of using assessment for summative purposes, i.e. assessment of learning; the improvement aspect is based on the idea of using assessment for learning purposes, i.e. assessment for learning; and the irrelevance aspect originates from the belief that assessment is useless or harmful to the efforts of both teachers and students, so it cannot be relied on (Harris, 2008). It has long been established in the literature that the successful implementation of assessment reforms in any education system is directly linked with how teachers view the role of assessment. Teachers’ positive view of assessment, i.e. using assessment for augmenting classroom pedagogies, results in productive assessment practices, whereas their negative assessment conceptions bring resistance or lead to the undermining of assessment reform policies and envisioned practices (Deneen & Brown, 2016; Fulmer et al., 2015).

In the current study, the participants’ belief in the pedagogical significance of various classroom assessments employed predominantly for monitoring student learning progress and their beliefs that teachers should have a strong role and responsibility in the whole process of developing and conducting assessments point to teachers’ conceptions of the purpose of assessment for *improvement* reasons. This is further highlighted by participants’ belief in establishing a strong formative assessment system and performing valid and reliable pre- and post-assessment analysis and evaluation procedures as two essential elements required for the improvement of overall assessment quality. These findings suggesting that teachers’ assessment conceptions are mainly inclined towards the *improvement* element are interesting especially considering previous research in this context, which reveals teachers’ summative view of assessment, i.e. the use of assessments for general accountability reasons (e.g. Almansory, M: EFL teachers’ beliefs and attitudes towards English language assessment in a Saudi university’s English Language Institute, Unpublished doctoral thesis; Almossa & Alzahrani, 2022; Umer et al., 2018). This indicates complexity in teachers’ assessment conceptions. Brown (2003) contends that teachers can hold manifold conceptions of assessment simultaneously as the structure of assessment conceptions is “multifaceted and interconnected” instead of simple and unvarying (p. 3). The findings of some previous research in different contexts (e.g. Gan et al., 2018; Hidri, 2016; Hui & Brown, 2010; Monteiro et al., 2021) endorse this argument. These studies find that although there exists a strong relationship between teachers’ conceptions of assessment for *accountability* and *improvement,* some teachers view assessment as irrelevant, which contrasts with the results of this study. Although participants’ assessment conceptions are predominantly inclined towards the *improvement* factor (see also Alonzo et al., 2021; Gebril, 2017), gaps in their understanding and awareness of some of the basic concepts of assessment reflect complexity because the successful implementation of classroom pedagogical and assessment practices requires teachers to be assessment literate.

Looney et al. (2017) postulate that the connection between teachers’ assessment conceptions and their assessment knowledge and practices is not straightforwardly enshrined in their assessment literacy; rather, it is multifaceted and highly complex. Since teachers’ assessment beliefs, knowledge and practice are shaped by a myriad of personal experiences and influenced by certain sociocultural and institutional policy dynamics, it is difficult for teachers to implement their beliefs and knowledge in practice without being influenced by these attributing factors. This is also true for teachers working in Saudi Arabia, which is a test-driven context with a tremendous emphasis on objective-type norm-referenced instead of criterion-referenced testing, and teachers generally lack opportunities to develop their assessment literacy (Latif, 2021; Umer et al., 2018). Considering the goals of Saudi Arabia’s Vision 2030, i.e. diversified economic, social and educational reforms, it is indispensable to reform teacher development programmes at the levels of policy and practice so that teachers can be trained to become assessment literate. Assessment-literate teachers are capable of ensuring student learning by translating their assessment conceptions and knowledge of effective assessment principles, i.e. validity, reliability, practicality, authenticity and washback, into effective practice (Alonzo et al., 2021; Brown & Abeywickrama, 2019).

The findings of the study have implications for policy makers, administrators and teachers in the process of LAL development for teachers. At the level of policy and administration, teachers’ LAL education and training programmes must take into account various contextual dynamics and needs as well as teachers’ personal assessment histories, beliefs and conceptions when planning and offering training. Additionally, the assessment purpose needs to be broadened by ensuring enhanced teacher participation in the assessment process, which is key to their assessment literacy development. More importantly, since teachers’ decision-making process regarding the implementation of assessments is directly linked to and arbitrated by the power relations around them, teacher agency must be recognized by ensuring enhanced teacher autonomy and a change in the teacher identity as self-assessors and critical pedagogues (Scarino, 2013; Xu & Brown, 2016). To address teacher assessment identity, it is equally important to consider “who teachers are in the process of assessment…as what they know and are able to do” (Looney et al., 2017, p. 15).

Focusing on teacher assessment beliefs, histories and personal theories in all teacher PD initiatives is vital, as these serve as mediational and filter tools that Xu and Brown (2016) term an “interpretive and guiding framework” for the theory-based knowledge passed on to them through seminars, workshops or lectures and its implementation. The acceptance or rejection of new assessment-related knowledge, ideas, policies or practices depends on their congruity with teachers’ conceptions of them. The alignment of teacher assessment beliefs with the principles of effective assessment practices, i.e. the assessments that prepare learners for lifelong skills needed to meet the needs and challenges of the modern era, is fundamental (Alonzo et al., 2021; Coombe et al., 2012; Looney et al., 2017; Popham, 2014). Linked to this aspect of teachers’ assessment identity is their sense of self-efficacy, i.e. the belief that valued targets can be achieved (Bandura, 1999). To ensure effective assessment practices, teachers’ self-confidence and belief in their ability to put their beliefs into practice are equally critical.

In short, taking into account some of the targeted educational reforms as per Vision 2030, that is, encouraging creativity and innovation in the learning environment; aligning curricula and instructional methods with contemporary trends; improving overall student values and equipping students with lifelong skills, there is a strong need to work towards a well-planned and sustainable view of teacher assessment literacy. Such a view moves beyond a strict focus on teachers’ assessment knowledge and skill development cognitively to their ability to negotiate diverse factors at micro, meso and macro levels having constantly situated and differential professional responsibility. Here, the micro-level focus refers to addressing teachers’ conceptions, beliefs, knowledge and experiences; the meso-level focus relates to institutional policies, classroom beliefs and practices; and the macro-level focus relates to assessment system-related values, protocols and practices (Fulmer et al., 2015). Without attending to the various dynamics of language teachers’ assessment identity in terms of their beliefs, conceptions, personal experiences, theories and self-efficacy at micro, meso and macro levels, LAL development initiatives for teachers cannot succeed.

Similar to many qualitative studies, the present study has some limitations. Miles and Huberman (1994) state that the quality of study data is determined by three factors: proximity to the data, actual behaviour and the checking of biases. In the present study, the data collection instrument was semi-structured interviews taking into account the research objectives. The use of classroom observations and document review as other methods could enable exploration of participants’ “actual behaviour” in terms of their assessment practices against the background of their beliefs, but this was not the study purpose. However, we acknowledge that since some of the participants belonged to our own work context and we worked closely with them in the research process, the research context involved bias. To control these personal biases as much as we could, we applied a number of techniques such as, using a sample with variety, using prompts and probes during the interviews and member checking (transcription verbatim as well as emerging themes). Despite these limitations, this study is critically important, as the findings may enhance our understanding of teachers’ implicit beliefs, values, preconceptions and personal theories regarding assessment, which are an essential aspect of their assessment literacy.

## Conclusion

The study aimed to explore various dynamics of tertiary EFL practitioners’ assessment literacy in terms of their beliefs, personal theories and conceptions that inform their conceptualizations, interpretations, decisions and judgement related to assessment practices. The findings suggest multiplicity, diversity and complexity in teacher assessment beliefs. The findings also reveal that although teachers believed in the use of assessment for improving the overall pedagogical process, reflecting positive assessment conceptions, there were gaps in their assessment knowledge base.

Despite the limitations of this study, the findings have important implications for policy making and teacher PD programmes for LAL development. Future research needs to further investigate language teachers’ assessment beliefs in terms of their assessment identity in diverse EFL/ESL contexts by exploring various factors that influence their assessment conceptions and practices. These factors include teachers’ motivation, self-efficacy, agency issues, demographic profiles and teaching beliefs as well as contextual dynamics. Moreover, considering the important role of other stakeholders, such as students and policy makers, in the assessment process, future research should also explore these stakeholders’ assessment beliefs and personal theories.

## Supplementary Information

**Additional file 1.** Semi-structured Interview Protocol.

**Additional file 2.** Sample of Participant Interview Transcript.

**Additional file 3.** Data Analysis – Initial Colour Coding – An Example.

**Additional file 4.** Data Analysis –Coding Procedures – An Example.

## Data Availability

The dataset supporting the conclusions of this article is included within this article and its supplementary files.

## Abbreviations

LAL: Language assessment literacy
EFL: English as a foreign language
ESL: English as a second language
TCoA: Teachers’ conceptions of assessment inventory
SRM: Selected response method
CRM: Constructed Response Method
PRM: Personal response method
