# Supplementary material for: Teacher beliefs, personal theories and conceptions of assessment literacy—a tertiary EFL perspective
Source: Lang Test Asia. 2022 May 2;12(1):11. doi: 10.1186/s40468-022-00158-5 (PMC9057650; doi:10.1186/s40468-022-00158-5)
Supplement: Supplementary file 1 — Additional file 1. Semi-structured Interview Protocol. [file 40468_2022_158_MOESM1_ESM.pdf]

Semi-structured Interview Protocol

1. What is your definition of a good assessment?
    - What are its characteristics?
    - What includes in it?
2. In your opinion, who should be involved in the conduct and design of assessment processes?

3. What do you understand by the term “classroom assessment”?
    - In your view, what are the things included in the classroom assessment?

4. What classroom assessments do you usually use in your classes?

5. How about the classroom assessments such as self-assessment; peer-assessment; portfolio-assessment; teacher-student conference; oral presentation; reflective journals; authentic assessment? Do you use any of these?
    - If yes, how often do you use each of these?
    - What influences your decision to choose them?

6. In your opinion, which of these classroom assessments is the most useful in assessing students’ learning? Why? Why not?

7. How do you usually assess your students’ receptive skills?
    - Which assessment methods would you like to use? Why? Why not?

8. How about productive skills? How do you usually assess them?
    - Which assessment item types/methods would you like to use? Why? Why not?

9. How do you understand the difference between holistic and analytic marking?
    - Which one would you prefer marking/ grading your students’ writing exams? Why?
    - Which one would you prefer marking/ grading your students’ speaking exams? Why?
10. In your view, how should the students’ results be interpreted?
    - How about the reporting and communicating of assessment results?
    - How should they be communicated to students? …to administrators?

11. In your opinion, how could the quality of an assessment, in general, be improved?
12. How do you ensure that your planned and designed assessment meets the quality standards?

Thanks very much for your time
