# Supplementary material for: Teacher beliefs, personal theories and conceptions of assessment literacy—a tertiary EFL perspective
Source: Lang Test Asia. 2022 May 2;12(1):11. doi: 10.1186/s40468-022-00158-5 (PMC9057650; doi:10.1186/s40468-022-00158-5)
Supplement: Supplementary file 2 — Additional file 2. Sample of Participant Interview Transcript. [file 40468_2022_158_MOESM2_ESM.pdf]

Sample of Participant Interview Transcript

Interview with Louise:

Researcher: What is your definition of a good assessment? I mean its characteristics or the things that make an assessment a good assessment.

Interviewee: I think a good assessment should be… not sure about the right word, but I think it should be such that challenges the students to think…it should not always be True/ False or MCQs-based... the questions should be of quality even if they are True/ False or MCQs-based…they shouldn't be simple and easy… they should force the students to think before they answer, so I think it has to challenge the students… not too much because you don't want to challenge them about something outside what they have learnt, but it should be based on the material that they have taken… thought about it and they are able to express it in their own words... I'm not saying that I don't agree with True/false/ MCQS-based…I mean I don't underestimate their usefulness but I don't think they should be obvious and simple.

Researcher: So, you mean that a good assessment is the one that challenges the students…. could you please elaborate on this?

Interviewee: A good assessment should be strong….I mean…it should be valid and fair. It should be based on the questions that are within the range of all level students…high achievers, mediocre, and low achievers…the test questions should assess the material or skills that the learners have been exposed to….and when I say that a good assessment should be fair, it means that…since you have students who are weak, strong and between, so probably the assessment questions should be directed towards who are in between so that the weaker students can ...well. can raise themselves up to the level of average ones and so on…

Researcher: So, in your opinion, who should be involved in the conduct and design of assessment processes?

Interviewee: I think it depends on the test … for example, you can consider the Preparatory Year Programme ... since the goal is to develop a level of English skills that are effective in an international environment, so that kind of assessment used at this level should be outside of a single university because they should be compared to English language speakers all across the world. so, I think at PYP level you can have some international exam TOEFL or IELTS or any Cambridge exam...any of those exams, but there should be a kind of some unified level across all these schools because of what they are doing.. For example, Japanese, if you want to learn to speak Japanese, you take a Japanese level test, or Arabic … any language because you are competing with other speakers of that language around the world. However, for the Bachelors level courses, it is different. For example, I teach Public Speaking, public speaking skills can change depending on what kind of department you are working in …but there are some general ideas that are the same, but still the course itself is mostly what you taught in that class… there is a degree of ability that needs to be there and it should be across many places, other colleges…

we talk about bench marking… being able to benchmark those courses against other universities to see if we have competitive graduates should be the goal…

Researcher: So, who should take the responsibility of conducting and designing an assessment … the course teacher, the institutional test committee or any external organization?

Interviewee: I think it depends on the subject. In general, I think it is better to have teachers who write the initial exam but having a committee who can look it over is essential because that committee can follow certain standards … for example, here at our work place, we have a committee that can look across courses of a certain level and see if all the students in that level are being tested…I mean if the questions align with the ability level of all level students… whereas course teachers only focus on their course, so to make sure that all the students are being tested at the similar level whatever that level is.. Senior, Junior, Freshman, Sophomore… and as regards the design of the assessment ... I think that it should also reside with the teacher because she/he knows how they have taught the students and what the understanding of the students would be...ah.. this reminds me of when I took the GRE exam, I think it was the first time I took it to go to the Masters Programme ... even though I'm a History Major, they asked me a question about Icons in Tribal Africa and I was supposed to write 5 paragraph essay... I went blank.. I couldn't think of anything... my History studies were in Islamic history, Judaism and Christianity… and the expectation was to write about African Tribes ... I went blank ... I couldn't think of anything. I had no clue, so I had to go back and take the GRE exam again...So, an assessment outside preparation can sometimes be very misleading…

Researcher: Okay…let's talk about classroom assessments…. in your opinion, what is "classroom assessment"… I mean what things/activities can be included in the classroom assessment?

Interviewee: First of all, classroom assessment is what is going on in the classroom at the time when the information is being conveyed to the students...and I think it can take many forms ... in my class, I always try to give students examples of test questions and ask them the answers... I also, try to let them ask questions and then I give them feedback ... I tell them the answers with the reasons ... I think classroom assessment should always be there even in a lecture class … the classroom assessment time is the time to ask them questions orally, so they can put their ideas together.. it is in the classroom where they can develop their critical thinking skills, so they can be able to produce the answer on a written exam... I think classroom assessment can also involve pop quizzes, short little things ... ah ... another teacher in another college actually encouraged me by … I was observing her class and she showed the test questions to the students on the PowerPoint ... that was such a good idea because there you have sample questions that the students can look at...

Researcher: So, if I ask you to name some of the classroom assessments that you usually use in your classes, what will you say?

Interviewee: Well, I use different classroom activities that are… I think classroom assessments depending on the course I'm teaching and the students I'm dealing with… I mean their level ...

mostly, I use presentations, short quizzes… etc. Also, there are a lot of question – answer sessions in my classes, so..yeh…different classroom assessments.

Researcher: How about the assessments like self-assessment; peer-assessment; portfolio-assessment; teacher-student conference; oral presentation; reflective journals; authentic assessment etc? Are you familiar with these?

Interviewee: Yes, I think… pretty much.

Researcher: How often do you use these assessments?

Interviewee: Self-assessment.. this is as I understand is to evaluate themselves on what they are doing. So I use this a lot; Peer-assessment - Sometimes only... Portfolio Assessment - I don't use it very much, but most of my courses are productive-skills oriented not receptive skills-oriented.... not writing, so I don't use Portfolio Assessment very much… Teacher-student conference and Oral presentation - these ones I use all the time; Reflective journals - I think we should use it all the time... I believe it is highly useful... but I never use it… Authentic assessment - I always try to use it ... even for the subjects like Phonetics and Phonology ... I try to make it reliable always ….

Researcher: So, how do you make your assessments authentic… I mean what is your understanding about an authentic assessment?

Interviewee: Well, I try to ensure reliability of the assessment…but...I am not sure…I think.. I don't know what an authentic assessment really means… but is it an assessment which is something reliable… this is what I feel...yes, I do if it doesn't have meaning more than this….

Researcher: What influences your decision to choose the above classroom assessments?

Interviewee: I think it depends on the content what we are doing in the class... for example, peer assessment, I especially use it in Conversation and Public Speaking because after a student speaks, I ask other students to evaluate that student… sometimes in a written form and sometimes orally…it is pretty hard to get students to say things orally, but I try to encourage that in the classroom.. For example, a student finishes the presentation, so I ask what was the best part of this presentation.. what did you like the most...and usually, I start with the positive.. and I try to reinforce.. they say e.g. I like the PPT colour.. yeh, GOOOD... I try to reinforce that ... and I never say what did the student do wrong.. I say.. what part of this could have been better.. they say but teacher it is perfect.. I say well, no body is perfect.. even your teacher is not perfect.. what can you do better.. I try to give the students an example.. look at this slide…

Researcher: What about teacher student conference? How do you use it?

Interviewee: In all my courses, I try to use worksheets/ handouts and when you are watching public speaking every day...when the students are doing the study questions on-line, I usually walk around and ask questions like ... which ones are wrong and why are they wrong? I try to

develop the critical thinking skills...but one on one trying to apply the principles we have learnt in the class.

In the Conversation class, they do a lot of pair or group work... they have to come up with agree and disagree statements about the topic.. so when they are doing that I go around and ask them questions about the sentences they wrote and so on…

Researcher: Ok.. what about Reflective Journals?

Interviewee: I think it is very important but I don't do it in the way it should be or could be done... for example, I teach public Speaking, so before the students speak about the Speaking Task, I always encourage them to write... I try to give them time to put ideas together, so in a sense, I think that's like a reflective Journal.... and then I evaluate what they did...For example, I ask a question: Fatimah, has the education of women in KSA has become any better or worse… give me a sentence or two...what you think about that.. after you give me the answer, I will give you my feedback about what you can improve, or I can ask you to elaborate on a certain point more. Since I have enough time to not only cover the content but give them/ and myself time to reflect...at the end of the class on what they learnt to assess themselves... they can adapt to that...sometimes with my comments and sometimes without my comments… look at it another time…go back to week one/two and let's see what you wrote…have you improved or gone down.. I think they can be and should be used a lot, but I don't use it a lot...

Researcher: In your opinion, which of the classroom assessments is the most useful in assessing students' learning? Why? Why not?

Interviewee: Because I teach Public Speaking and Conversation, so Oral Presentations is the most useful… it is only because I teach Public Speaking... In fact, Presentations is the goal of the course... they need to be able to express themselves orally in order for them to lose their fears and be able to speak...we need to give use Oral Presentations as the assessment...they need to be exposed to as many speaking opportunities as possible.. in general, in my opinion, self and peer-assessment, and reflective journals can be used with great learning benefits in the class… authentic assessment is also very useful…if you ask me to select the one which is the most useful… I would say… peer-assessment as this would improve students' confidence, critical thinking and evaluation skills.

Researcher: How do you usually assess your students' receptive skills? I mean… Listening and Reading skills… Or in your opinion, how should they be assessed?

Interviewee: I usually use a combination of MCQ-based and short answer type questions to assess Reading and Listening skills, but my favorite would actually be short answers-based questions, because I feel that I can reflect.... and the students have an opportunity to construct responses and express their ability to express what they are learning.. I think this assesses their critical thinking skills the best.

Researcher: How about the productive skills? Which assessment item types or methods would you like to use for assessing them?

Interviewee: I usually use short answer and essay type questions to assess Speaking and Writing skills…. but I think it is the Personal Response method what they are doing in the class when they give a speech… but not really because even in Public Speaking and Conversation, they are giving a form.. some sort of thing that they are putting in place.. for example, I tell them… here is the outline… go fill in the outline.. they have specific things… for example, in Intro.. they have to include a greeting… it has to include credibility… it has to include background information... it has to have a central idea…so, they have to… sort of short answer these things..

Researcher: How about marking/ grading assessments…what is your understanding about the difference between holistic and analytic marking? Which one would you prefer marking/ grading your students' writing/ speaking exams? Why?

Interviewee: Holistic Marking means looking at the whole exam as a whole idea to get general idea, whereas analytic marking means marking each & every response individually, and I would prefer to use Holistic marking for Writing all the time because we face issue where a student has really great content and very poor language skills. Well, we all know that if their lang. skills are too poor, it affects the content... however, if they have very good language skills, usually, their content is very good, so…in that case, I have no problem in holistic marking … I don't need to mark every single thing wrong in a student who produces very well.

As regards Speaking exams, I think they have to be graded holistically.. I don't think there is any way to grade them analytically... on the other hand, I create a rubric that looks for different parts… like as I said before, in the Intro. ..there are certain things you have to include, so I check for those things, but in general, I look at it in a holistic way because if they put everything required in the Introduction, it will probably make sense; it will probably be a very good intro. but if there are missing parts, they will be choppy and won't make sense..

Researcher: The rubric that you prepare to mark your students' assessments, do you share it with the students before the assessment? Why/Why not?

Interviewee: Always because I think it is very useful. It tells them that if their performance is not aligned with the different aspects of the rubric which are based on what they have been taught throughout the semester, they lose marks… and students tend to focus on marks. By giving them rubric, I feel that I draw their attention away from marks... Also, for example, in Public Speaking, when I want them to speak for ten minutes, and students are like...ahhh.. how can I speak for ten minutes.. but when you break into pieces like that.. e.g. Intro has four parts, so each part will last for a minute or half a minute ... so covering all parts helps them to finish the task using ten minutes... they don't feel how the time passes, so they don't take it as a difficult task...

Researcher: In your opinion, how important is post-assessment feedback?

Interviewee: I think post assessment feedback is imperative… I really feel so because here at our work place, we don't give any feedback to the students after the final exam... we must not forget that the final exam is 40 % of their final grade and they don't receive any feedback on

that... and I think that we are doing our students injustice by saying so much that their grade is important but not important enough to give them any feedback…ah.. I wouldn't do that this way if I would be given the option.

Researcher: Do you use individual or group feedback? Which one do you find more effective? Why?

Interviewee: I think both individual or group feedback are important because… but I think that depends on the learner… for example, individuals who are poor need specific feedback to address their personal needs to improve…I think group feedback has its benefits when everyone is making the same mistakes.. When most of the students have missed a question or their answer is wrong in that question, I know that there is something wrong with my teaching… you know maybe I didn't explain it right … maybe all the students were in a different world because they just had a difficult exam in another class… but for some reason that concept is not clear and that also tells me that I need to teach that concept again ... and put it on another exam in the future so that we can see if they grasped the concept... I think that is a big part of group feedback by looking at how a group performs ... whereas individual feedback caters to the needs of individual students and addresses individual problems…

Researcher: Can you give me some examples the way you give feedback? What is your way of giving feedback?

Interviewee: I have a senior student who had to repeat some courses again and again and she has been here for the last six years ... and on her midterm, she gave a speech on Dried Fruit.. there was nothing wrong with the topic but that there was no depth in it.. so I pulled her aside after the mid-term exam and I sat with her and explained the difference between depth and her speech; it was superfluous from the perspective of ideas… I told her that she needs to develop her speech by giving some facts about nutrition ... there are plenty of facts about dried fruits, so you need to develop more specific resources… more scientific resources... I asked her to take the printouts.. I sat with her and highlighted certain sources that she could add ... I told her that If you change your speech by adding facts with examples, your speech will become persuasive… and in her case, I will take whatever she did and encourage her to develop it as much as she can because she is week. She doesn't need to start with a brand new topic and new ideas…she can take this idea and develop it....here we don't have any mechanism of retaking the mid-term based on the feedback of the teacher.. I don't mind if the students are allowed to retake the mid-term and develop… I don't think it is bad... the end goal is the students' learning progress and overall development...

Researcher: In your opinion, what students’ assessment records are important to maintain? What various methods do you employ to record your students’ achievement?

Interviewee: Actually, I think students' assessments need to include all these different things we have already spoken about like self- assessment, peer assessment, portfolio assessment, Reflective journals in every class and I think all of these things are important and I personally keep a notebook or a folder for each class and I have the attendance list and I have a code on the attendance list… and on there I note things like who has the book and who doesn't…I keep track

of who is always wanting to go out … to the toilet/ cafeteria...I keep track of who is always caught on her cell phone…I understand… there are some emergency calls… I don't keep note of those but things like behavior.. I do keep track of that… occasionally; it happens that a student is sleeping… I keep track of that and see if it is happening consistently or… I keep these notes about each and every student… it is time-consuming, but I have been doing it for so long now that it somewhat natural... Also, if a student says that she has a birthday today, I keep a note of that and ask that student in the next class if she did something… just in order to make some personal connection… ok tied in with assessment… if I have an idea about my students where they are personally, how they are developing or not... then when I work on their quiz/ mid-term results, I can make connections between them...the student who is more troubled is probably going to struggle with exam results…this is natural… so, I want to make note of that and try to encourage her by bringing some chocolates/ cookies in the class… something to make them more comfortable.

Researcher: In your view, how should the students' results be interpreted?

Interviewee: What do you mean here by interpretation…there are many ways you can interpret the students' results… it depends on the individual teacher…

Researcher: So, what is your way of interpreting students' results in a particular assessment?

Interviewee: Well, I use varied methods… sometimes, I try to evaluate whether my students are actually up to the standards...among other students...how they are going... how they are actually doing in relation to some particular skills... so for me results would be very important not just for giving them the mark...it is very important from the perspective of research as well. ...because it is one way of identifying strengths and shortcomings of the program too...let's say... For example, our case here... our students are actually at par with other institutional students.. by looking at the results of the examinations and the level of the students based on their performance, we can draw comparisons… so in that way we have a way of getting useful information as to how we are performing as an institution...

Researcher: In your opinion, what could be the most useful method of interpreting students' results… I mean… from the perspective of overall educational improvement?

Interviewee: Well. the main focus should be on the students' progress…whatever method you use, the ultimate goal should be …if your students are progressing in terms of learning or not…. though I am not sure if it is possible in every context… take our context here as an example….well...here, we are dealing with 25 to 30 students... ideally I would like to deal with them individually and see how they are progressing and work with each of them, but that's not the case because we are dealing with 30 students… so it is difficult… to deal with the results of the exams for each individual student's learning benefit…the function of the teacher to work with each individual student for his or her progress has been taken away from us...because we are dealing with too many students for a short span of time,... but sometimes I focus on those who are not achieving so well, and I'm actually excited and challenged to let them move to the higher level... maybe it is not fair for the better ones or the average ones because you have less time for them and your mind is set more towards those who should be achieving... because they

are behind...so, mainly it is because of a number of students that we are not able to deal with each and every student individually and look at their progress because as a teacher I would like to see their progress and not actually if they are getting high in relation to other students...

Researcher: How about communicating and reporting results to students and administrators… in your opinion, how should it happen?

Interviewee: Well, I appreciate the fact that we have a good system here...they have a way of generating the results data which instantly gives a complete report to show to the admin. that this is how our students have performed in the exams...hopefully, we can also do that in our classrooms as teachers...we can generate results data for our classes sake, too... but all we can actually do is to have results of the everyday assessments we are doing... well, feed backing to the students, as I have said, would be in the form of letting them know the general observations because we can’t do individual progression check... ideally, each individual student should be informed about his/her strengths and weaknesses…the points where they have shown improvement and the points that they still need to work on….

Researcher: So, if I ask your opinion about the ways to improve the quality of an assessment, in general… what would you say?

Interviewee: I think having a test committee that is responsible for reviewing the assessments before they are administered seems logical to me. Also, I like the idea of having an external reviewer… I think the way we do our exams, having another opinion.. I have even been known to hand my exams to my husband or my son when he was here and have them read the questions because they don't have any idea of what I'm teaching, but they should be able to understand the questions and understand what I'm asking the students to do. If they can do it, I can expect my students to do, too…they should be able to understand the questions and understand what they are expected to do… so, I think feedback from other people even those who are not of your field is important.

Researcher: How do you ensure that your planned and designed assessment meets the quality standards?

Interviewee: Well, I have some standards that I follow to ensure that my assessments meet them. These standards I have developed over my teaching experience. For example, while back we decided here in this college that all multiple choice questions will have four or more choices, but I always did that before because of experience in teaching.. I learnt over time that it is too easy to do a fifty fifty question (two options) or even a 33-33-33-or 34.. (three options) whatever...you can't...so, I am for four or more than four options in all MCQs-based questions…also, my biggest goal is, I guess, critical thinking… developing students' critical thinking, and even in these selected-response method questions, there needs the application of some level of thinking, so I try to avoid simple questions... Also, one thing that I always try to make sure is that the beginning questions are more easily answered to encourage students when they start taking the test...oh.. I know everything… so, simple one or two simple questions at the start... and at the end… I always try to end with a question that I feel the students can handle comfortably... even if it is writing… something that feel like --oh wow! I did this well and they

walk away from the exam with a good opinion instead of feeling that ...oh… I'm a miserable failure.

I think when our college started getting strict on exams and placed some standards, I already knew those and I was implementing them in my classes… these I learnt with experience.. I think there should be appropriate clear assessment quality standards at the institutional level...

Researcher: In your opinion, what is a fair assessment?

Interviewee: A fair assessment will also take into account an individual student learning ... or individual student ability… earlier we talked about a student who was struggling with the course, you know the one who did the speech/ presentation on dried fruit... because the rubric.. as I said I have these parts.. and… because she was able to do all the parts, so I was able to give her a fairly good mark for her mid-term,, but still she was required to show me that she met the criteria set... however, I am not going to compare her with other students in the class. She has her own needs, so she should be dealt with accordingly...

Researcher: Thank you very much for your time

Interviewee: It is a pleasure!
