# Supplementary material for: Teacher beliefs, personal theories and conceptions of assessment literacy—a tertiary EFL perspective
Source: Lang Test Asia. 2022 May 2;12(1):11. doi: 10.1186/s40468-022-00158-5 (PMC9057650; doi:10.1186/s40468-022-00158-5)
Supplement: Supplementary file 3 — Additional file 3. Data Analysis – Initial Colour Coding – An Example. [file 40468_2022_158_MOESM3_ESM.pdf]

Initial colour coding of the interview data (An Example)

Example 1.

Q.3 What do you understand by the term "classroom assessment"?
In your view, what are the things included in the classroom assessment?

Participant 1

…generally we start with asking some questions about a lesson without letting them know…this means that you are already doing some kind of assessment … that is a pre-lesson assessment or a kind of diagnostic assignment… it could be done orally or it could be in written form... just to get an idea how much your students have in terms of stock knowledge...or information… prior to the lesson... then through the lesson, you can do a variety of activities or tests that would also challenge their understanding of the lesson… it's like giving them. For example, questions/ answers; listing; diagramming. Some activities throughout the lesson...

Participant 2

Well, a lot of things can be included in the classroom assessment.. always we depend on the teacher in order to be the assessor of the students, but sometimes, the students themselves could be the assessors of themselves in peer review… for example, you can have two students sitting next to each other, talking to each other, and assessing each other, so it is not only the teacher but the students are also involved in assessing each other... similarly, you can have the groups that can have the assessment. In the groups they will be assessing themselves… of course, if we are talking about formal assessment, paper-based assessment, in this case, of course, we are going to use papers, so it is the teacher who will be the assessor, but still even if you are conducting a formal assessment, you want to be less formal, you also include the students in this kind of assessment by having them correcting each other's papers.

Participant 3

To me, any strategy we use in the class to monitor students' performance and progress is classroom assessment. Yeh, there can be multiple ways you can assess sts' learning inside the classroom but that depends on what the learning outcomes are in terms of the instruction being given- we can go for informal checks for understanding like questions in the class all the way to more performance type activities such as doing a short discussion/ presentation that's for speaking skills.. if it is the writing skills, perhaps they can do a practice written activity to see how they are doing on a particular aspect of writing.. I think the same can be done for all skills- we can have short mini assignments and mini assessments which we can do in class.

Pink: Definition of assessment Green: assessment strategies Blue: use of assessments
Yellow: consideration of course learning outcomes Grey: Contextual dynamics

Example 2

Q.6. In your opinion, which of the above classroom assessments is the most useful in assessing students' learning? Why? Why not?

Participant 1

Well, all these are useful and can be used with good results. However, as per my understanding and experience, it is the authentic assessment, which is the most useful, in assessing students' learning especially the slow learners ... but supposedly authentic learning... because I'm teaching in a school in the evening for a special program... for example, if our lesson is about food or restaurants… every week one day we go out to the restaurant and they order food in English based on what we discussed in the class.. for me, this is authentic learning.. it is real because they apply the vocabulary and expressions they learnt in the class, they could feel the meaning and value of the words and expressions they learnt in the class... that's very meaningful...any lesson which is meaningful becomes memorable...but, of course, it is not useful in every setting...for example, any setting that has institutional constraints in terms of using authentic assessment effectively, it is not useful... for example, in our setting, there are some issues, so I can't use it... because it is not required for us to go outside or bring many things inside... so, the problem is the environment... in these, self & peer-assessment, I consider as the least effective...because… for example, self-assessment... when at the end of the lesson, you ask the students if the particular concept covered in the class is clear to them, they all say" yes", but when you ask them questions later on, on many occasions, many can't answer... so I believe that self-assessment is not reliable...same is the case with peer assessment... for example, I tried it last semester... I selected five students to help me assess the presenter, and I noticed when they are close to the presenter, even if the presentation is very poor, they gave high marks… so, it I think it is not reliable.

Participant 2

Because I teach Public Speaking and Conversation, so Oral Presentations is the most useful.. it is only because I teach Public Speaking... In fact, Presentations is the goal of the course... they need to be able to express themselves orally in order for students to lose their fears and be able to speak...we need to give them Oral Presentations as the assessment...as many opportunities to speak as possible…

Participant 3

Well, I believe all are important and can be used... but they are used in progression, so they have a hierarchical significance...like, for example, during the beginning of the lesson, we just do self-assessment and peers assessment...we can't have oral presentations right away… because it is the beginning of the semester; they have to do the simple assessments first but towards the end, or as we go through...when you feel that the students are more confident, they have more knowledge of self-assessment, or they are more skilful, then we can use reflective journals or authentic assessments...

Pink: Definition of assessment Green: assessment strategies Blue: use of assessments
Yellow: consideration of course learning outcomes Grey: Contextual dynamics
