# Supplementary material for: Teacher beliefs, personal theories and conceptions of assessment literacy—a tertiary EFL perspective
Source: Lang Test Asia. 2022 May 2;12(1):11. doi: 10.1186/s40468-022-00158-5 (PMC9057650; doi:10.1186/s40468-022-00158-5)
Supplement: Supplementary file 4 — Additional file 4. Data Analysis –Coding Procedures – An Example. [file 40468_2022_158_MOESM4_ESM.pdf]

Coding Procedures_An Example

Themes	Sub-themes	Codes	Examples
Beliefs about assessment and testing in general	Varied views and interpretation regarding the characteristics of a good assessment	Reliability, validity, fairness, context-based	*"…basically, a good assessment should be first reliable, then valid and also fair. Moreover, it should be from their milieu that is inside of what their life is…I mean it should be context-based, too" (Ahyam)*
		Application of the learnt material<br><br>Production of the language	*"… a good assessment to me is the one that challenges the examinee to apply what he/ she has learnt; it gives the examinee an opportunity to write or produce what he/ she has learnt…" (Turki)*
	Importance of teacher role and responsibility in the design and conduct of assessment	Design and conduct of an assessment with the course	*"I think it is better if the teachers teaching the course design and conduct an assessment because they are in a better position to ensure test validity and reliability not an external body, which is not familiar with*

	processes	teacher<br><br>Test validity & reliability<br><br>Familiarity with the context	*the ntext…"(Ahmad)*
Beliefs about classroom assessment	Activities considered as classroom assessment	Multiple things<br><br>Students as assessors in peer-review (peer-assessment)<br><br>Questioning and answering<br><br>Surprise short quizzes<br><br>Projects<br><br>Assignments	*"…well, a lot of things can be included in the classroom assessment; besides the teacher, the students could be assessors of themselves in peer-review." (Ahyam)*<br><br>*"…In my opinion, whatever is going on at the time when the information is being conveyed to the students is classroom assessment; it can take many forms such as question-answers; pop up quizzes, assignments, projects…etc" (Karen)*

	Use of diverse classroom assessment activities for assessing students' learning	Varied activities as assessment<br><br>Assessment purpose<br><br>Course<br><br>One-on-one interviews<br><br>Presentations<br><br>Self-assessment<br><br>Peer-assessment	*"…Well, we can use a variety of assessments in the class depending on the purpose and the course; as regards me, sometimes, I have one-on-one interviews, other times, we have presentations, then sometimes I ask students to check their own and their peers' work…so a lot of things…" (Ahmad)*
	Different reasons for using classroom assessments	Content<br><br>Peer-assessment	*"I think it depends on the content what we are doing in the class, for example, I use peer-assessment a lot in my Conversation and Public Speaking classes; after a student has spoken, I ask others to evaluate her….and so on" (Karen)*<br><br>*"I think it is the peer-assessment that probably I have found the most*

	Effective activities in different classroom contexts	Peer-assessment<br><br>Good for both receptive and productive skills<br><br>Oral presentations<br><br>Good for assessing all skills<br><br>Assesses production of the language	*useful…; I use it in case of both receptive as well as productive skills" (Ahyam)*<br><br>*"Well I find oral presentations as the most useful in terms of assessing all skills because it assesses production…" (Ahmad)*
Beliefs about assessment methods, strategies and procedures	Methods for assessing macro skills	<u>Receptive Skills</u><br><br>True/ False; Matching; MCQs<br><br>Understanding of the language not production<br><br><u>Productive Skills</u><br><br>Short answer type questions<br><br>Essay type questions	*"…well, for receptive skills, I normally use True/ False, Matching, MCQs as they are good for students' response to language…their understanding, whereas for productive skills, I like to use short answer/ essay type questions…"*

			(Ahyam)
		Productive Skills<br>Self & peer-assessment<br>Portfolios<br>Conferencing<br>Capacity to deal with students individually	*"… I think for productive skills, I would like to use self & peer-assessment, portfolios and some kind of conferencing because this lets you deal with students individually"*<br>(Ahmad)
	Preferred marking approach based on the assessment	Formal Exam<br>Writing & Speaking<br>Holistic marking/ grading approach	*"Well, I would prefer to look at the whole writing exam essay holistically to get its general idea and mark it- the same I will do for the grading of the Speaking exam; I don't think there is any way to grade Speaking analytically"*<br>(Karen)
	Importance of post-assessment feedback	Post-assessment feed backing<br>Essential for learning	*"…I believe feed backing after an assessment is essential for learning; I do both individual and group feed backing*

		Individual as well group feed backing approach<br><br>Time availability<br><br>Class size	*depending on the time at my disposal and the class size" (Daniel)*
Beliefs about assessment results and record keeping	Interpretation: criterion-referenced preferred<br><br>Varied approaches to communicating results to different stakeholders	Large class size<br><br>Not enough time to see students' learning progress individually<br><br>Given the choice, preference for keeping track of students' progress individually as per course ILOs<br><br>Communicating results to students<br><br>Different approach depending on every individual case-<br><br>better achievers/ low	*"…well, here, we are dealing with 30 to 35 students in 6 teaching weeks for one CEFR level-based course, so we are not able to deal with the students individually and see their progress; ideally as a teacher, I would like to see every individual student's progress in relation to course ILOs not if they are getting high in relation to other students in the class…" (Daniel)*<br><br>*"…for students, my approach is basically case to case basis…for example, if they have low marks, they don't want their results to be*

		achievers	*communicated in front of others; however, better achievers want their results to be announced in public…so, it depends…" (Alfonso)*
	pedagogical significance of record keeping	Importance of keeping track of students' learning progress<br>maintaining portfolio<br>Evidence related to students' learning progress- important	*"…I think keeping assessment-related records is certainly important …teachers should definitely maintain portfolio or some form of evidence of the students' progress…it is very important for the teacher as well as the students" (Turki)*
Beliefs about assessment quality standards	Importance of formative assessment system-based procedures<br><br>Pre- & post	Continuous classroom assessment- based system<br>Key to improving assessment quality	*"….to me, it is the system of continuous assessment that you do every day in your classes to assess your students' progress that is key to improve assessment quality…." (Ahmad)*<br><br>*"I think having a*

	assessment analysis and evaluation-essential	Test committee<br><br>Pre- assessment validity checks<br><br>External reviewer<br><br>Post-assessment validity and reliability checks	*test committee responsible for reviewing the assessments for validity before they are administered seems logical to me; also I like the idea of having an external reviewer for the post- exam validity and reliability….” (Karen)*
	Personal quality checks related to self-designed tests	Questions based on context-based materials<br><br>Assessing critical thinking skills	*“Well, my focus is primarily the students that I ‘m dealing with, so my questions in the exam are contextualized meaning to say that they are based on the material that they can relate to. Also, my goal is their critical thinking skills, so I design questions accordingly… (Daniel)*
	Varied views and interpretation of test fairness	Importance of considering individual student learning and ability	*“For me, a fair assessment will take into account an individual student learning; the one that is designed*

		level	*taking into consideration every individual student's ability level…" (Karen)*
		Teacher objectives<br>Purpose of assessment<br>Assessment reflecting their true ability level	*"…I think a fair assessment should be based on my objectives as a teacher…also, it should be something that my students need; it should give me the results that I feel reflects what I want to know in terms of their level…." (Daniel)*
		Students involvement in the assessment process<br>Importance of students' voice	*"…in my opinion, to have a fair assessment, student involvement in the assessment process is important; I mean whatever basically can give them a chance to have their say…" (Turki)*
